# Supplementary material for: Strategic Template Filtering Accelerates Fragment-Based Peptide Docking
Source: J Chem Inf Model. 2026 Jun 16;66(15):9317–27. doi: 10.1021/acs.jcim.6c00902 (PMC13471421; doi:10.1021/acs.jcim.6c00902)
Supplement: Supplementary file 1 [file ci6c00902_si_001.pdf]

Supporting Information for Publication

Nirit Trabelsi-Mescheloff*[1], Julia K. Varga*[1,2], Alisa Khramushin[1,3], Sergey Lyskov[4], Ora Schueler-Furman[1*]

1 Microbiology and Molecular Genetics & Quantitative Molecular Medicine, The Hebrew University of Jerusalem, Jerusalem, Israel, 91120.
2 Biomolecular Sciences,Weizmann Institute of Science, Reẖovot, Israel, 76100.
3 Institute of Bioengineering, École Polytechnique Fédérale de Lausanne, Lausanne, Switzerland, 1015.
4 Department of Chemical and Biomolecular Engineering, Johns Hopkins University, Baltimore, Maryland, United States, 21205.

* Email: Ora.Furman-Schueler@mail.huji.ac.il

Supplementary Figures

- **Figure S1**: Comparison of performance on the LNR set (n=39) of the original PatchMAN to PatchMAN2 with BSA filter
- **Figure S2:** Performance for the PFPD set (n=23) based on DockQ measures(original PatchMAN vs PatchMAN2 with BSA filter)
- **Figure S3:** Masking interfaces of obligatory homomultimers
- **Figure S4:** Masking reduces the number of extracted patches and fragments
- **Figure S5:** DockQ docking performance before and after masking
- **Figure S6**: Calibration of filtering thresholds on the PFPD set
- **Figure S7**: Calibration of number of refinement runs and decoys selected for clustering
- **Figure S8.** Scatter plot comparing docking performance obtained with $\text{PatchMAN2}_{\text{baseline}}$ and PatchMAN2 Focus on the PFPD and LNR benchmark set
- **Figure S9:** Comparison of receptor surface area covered by hotspot-defined and focus-defined input regions.
- **Figure S10:** DockQ values for PatchMAN2, Focus and Hotspot results on the PFPD set
- **Figure S11:** PatchMAN2 Focus, DiffPepDock and RAPiDock results on the PFPD set
- **Figure S12:** PatchMAN2 and AF3 results on the PFPD set
- **Figure S13.** Docking performance PatchMAN2 and AF3 on novel interactions

Supplementary Tables

- **Table S1**: Benchmark sets used in this study
- **Table S2**: Definition of BSA cutoff for different peptide lengths
- **Table S3**: Runtime comparison across CPU nodes
- **Table S4:** Performance of PatchMAN after addition of BSA filter
- **Table S5**: Masking the obligatory interface of homomultimer complexes improves PatchMAN results
- **Table S6**: Effect of focus filters on performance (PFPD set)
- **Table S7**: Performance of PatchMAN after addition of focus filtering
- **Table S8:** Results of global runs of $\text{PatchMAN2}_{\text{baseline}}$ and AF3 on a set of 8 novel peptide-receptor complexes.

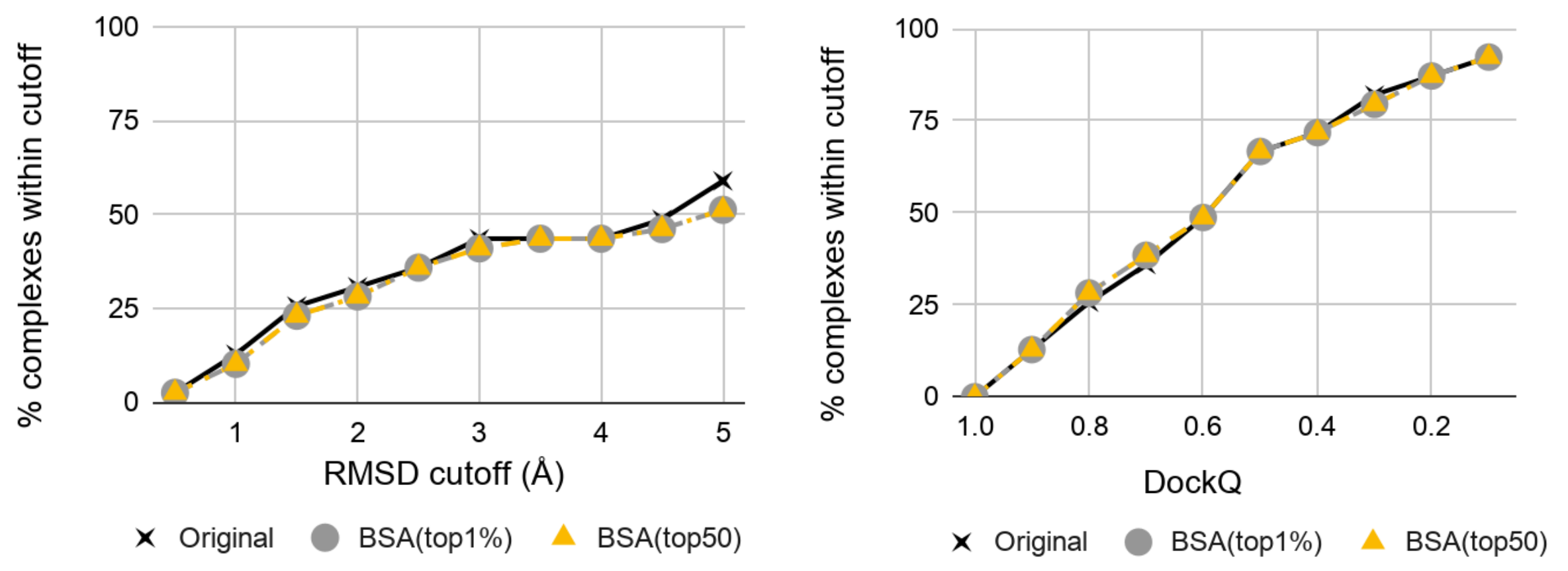

Figure S1. Comparison of performance on the LNR set (n=39) of the original PatchMAN to PatchMAN2 with BSA filter (clustering the decoys using either the top 1% [BSA(top1%)], or *max(top 50, top1%)* [BSA(top50)]. BSA(top50) is the baseline for the following analysis ($\text{PatchMAN}_{\text{baseline}}$). Legend as in **Figure 1E. Left:** Performance measured using RMSD**. Right:** Corresponding performance based on DockQ values**.**

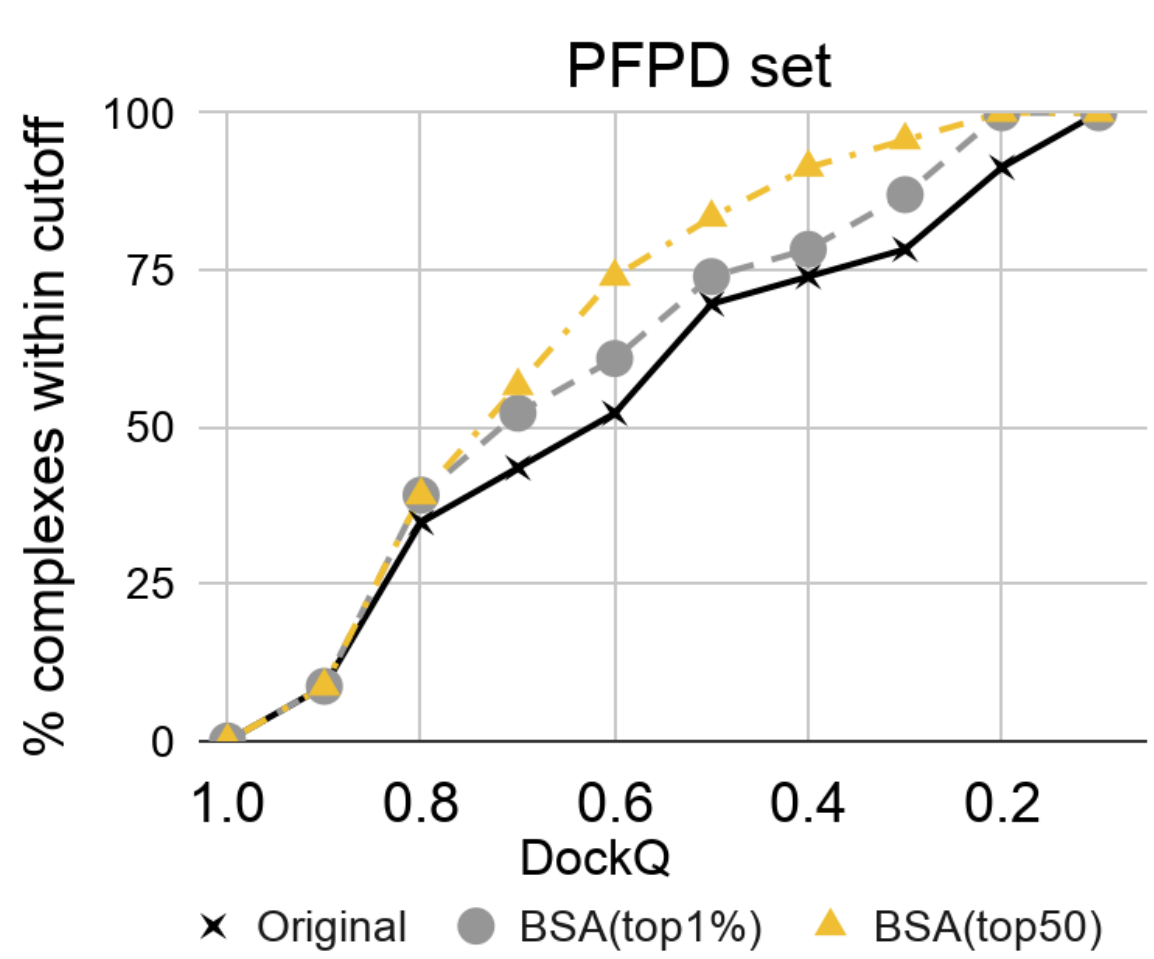

Figure S2. Performance for the PFPD set (n=23) based on DockQ measures. Legend as in **Figure S1. Accompanies Figure 1E.**

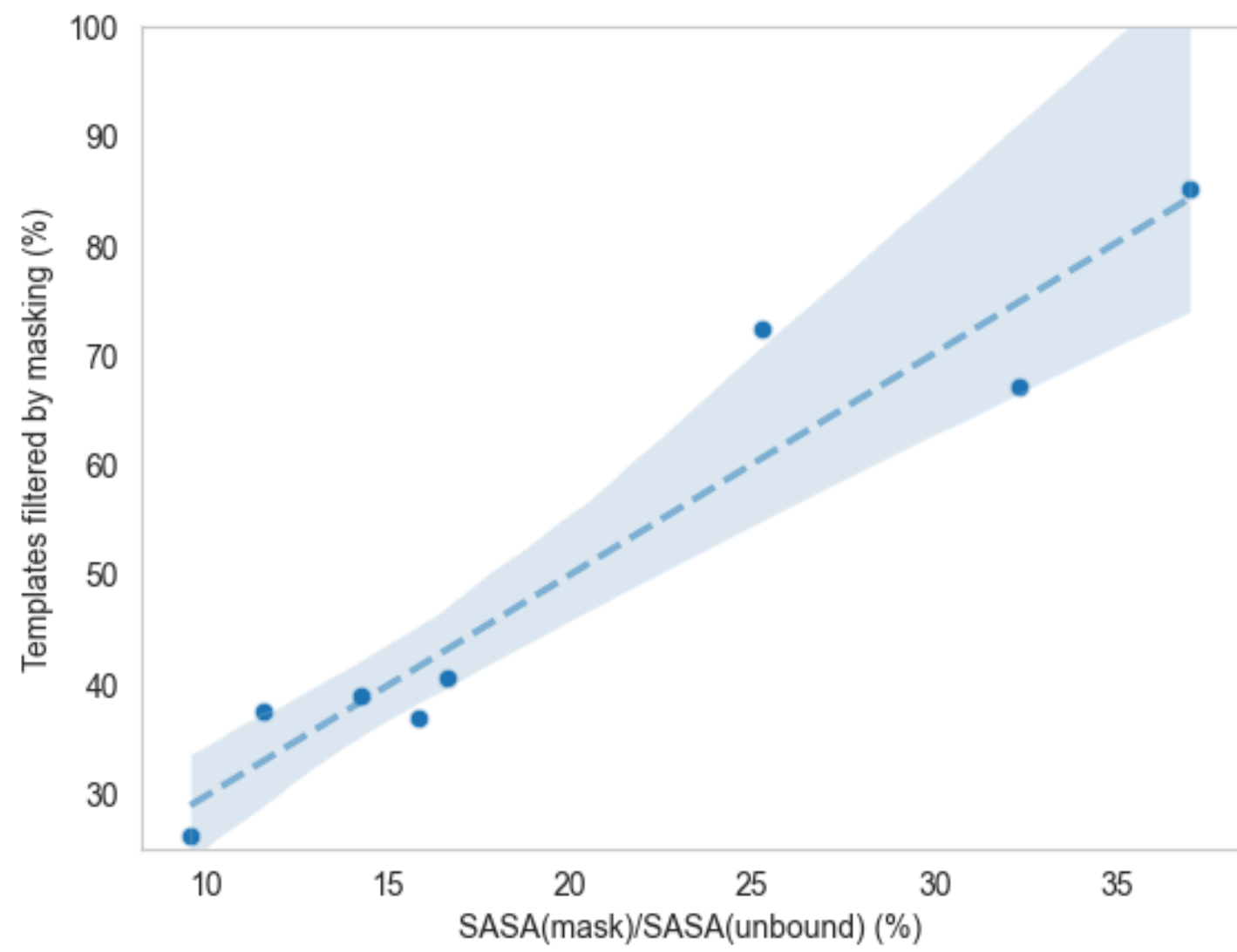

Figure S3. Masking interfaces of obligatory homomultimers. Masking one third of the surface can result in the removal of 80-90% of the candidate fragments, due to the popularity of the interface for binding. The percentage of fragments filtered by masking is correlated with the percentage of the receptor surface covered by the multimer interface. SASA - Solvent Accessible Surface Area.

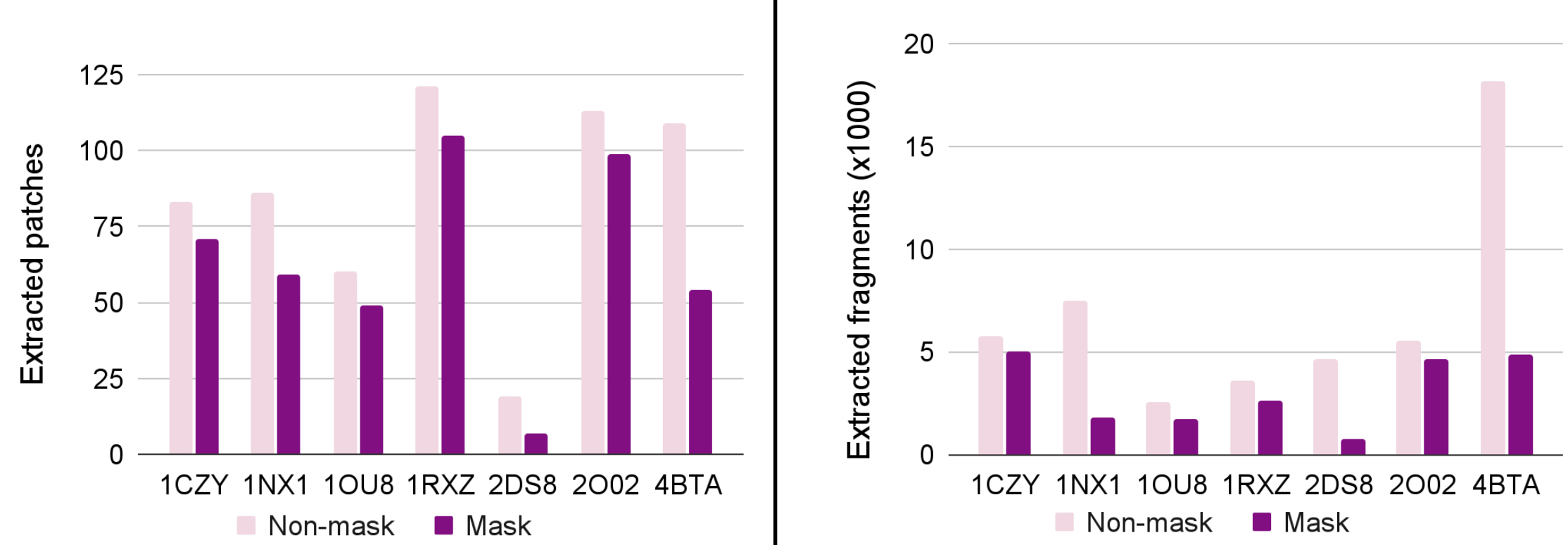

Figure S4. Masking reduces the number of extracted patches (**left**) and fragments (**right**).

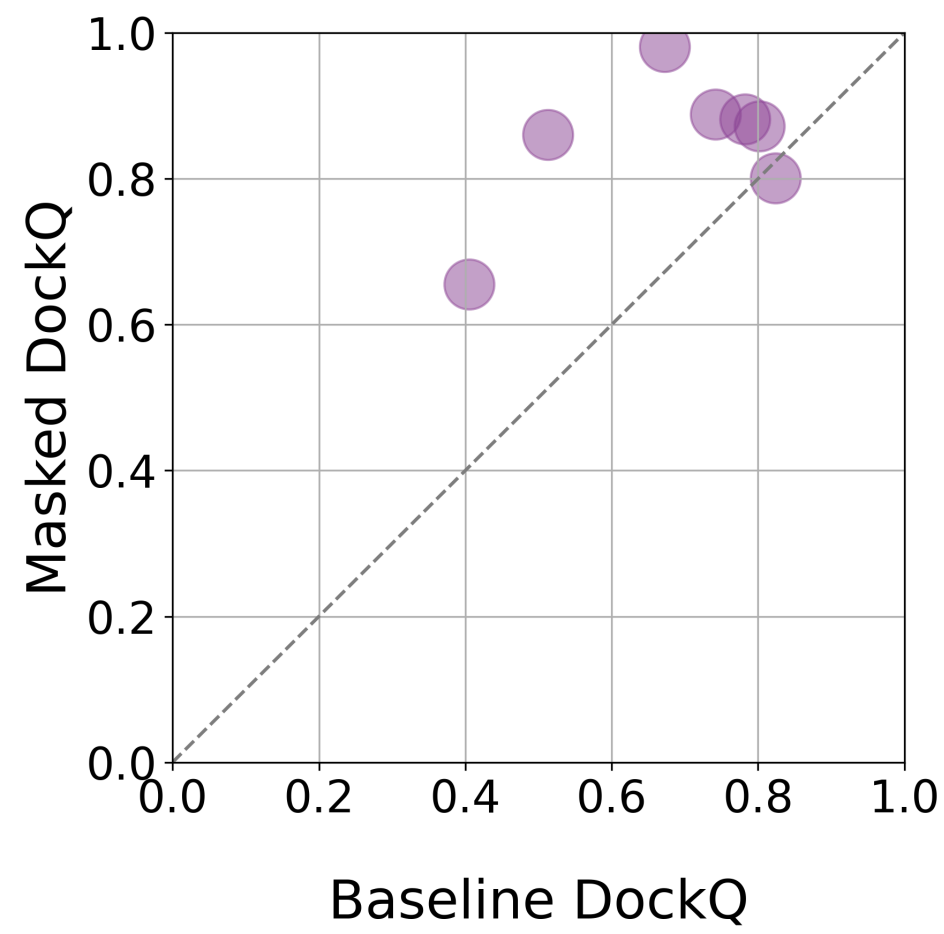

Figure S5. Docking performance before and after masking, measured by DockQ. **Accompanies Figure 2B.**

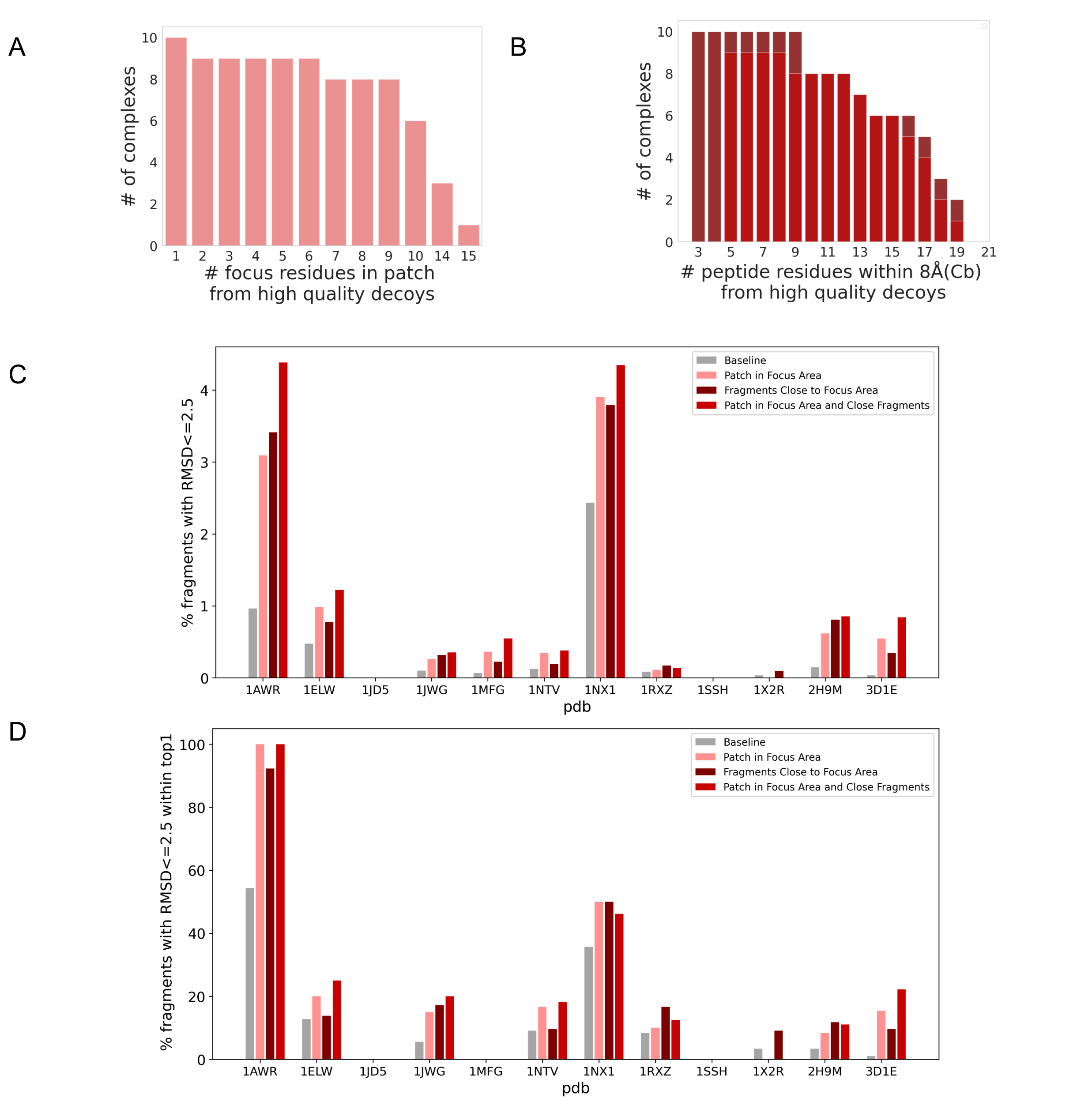

Figure S6. Calibration of filtering thresholds on the PFPD set. **A.** Distribution of the number of overlapping residues between the focus area and patches producing successful models. **B**. Distribution of the number of focus residues contacting fragments that produce successful models (RMSD within 2.5 Å after their refinement)**. C-D.** Effect of focus filtering on fragment selection. Filtering enhances the selection efficiency of accurate models within the top-ranked predictions for the PFPD benchmark set. **C.** Fraction of fragments that produce successful models. **D.** Fraction of fragments that produce final successful models (*i.e.*, among the top 1% retained for the final clustering step in PatchMAN). **Accompanies Figure 3**.

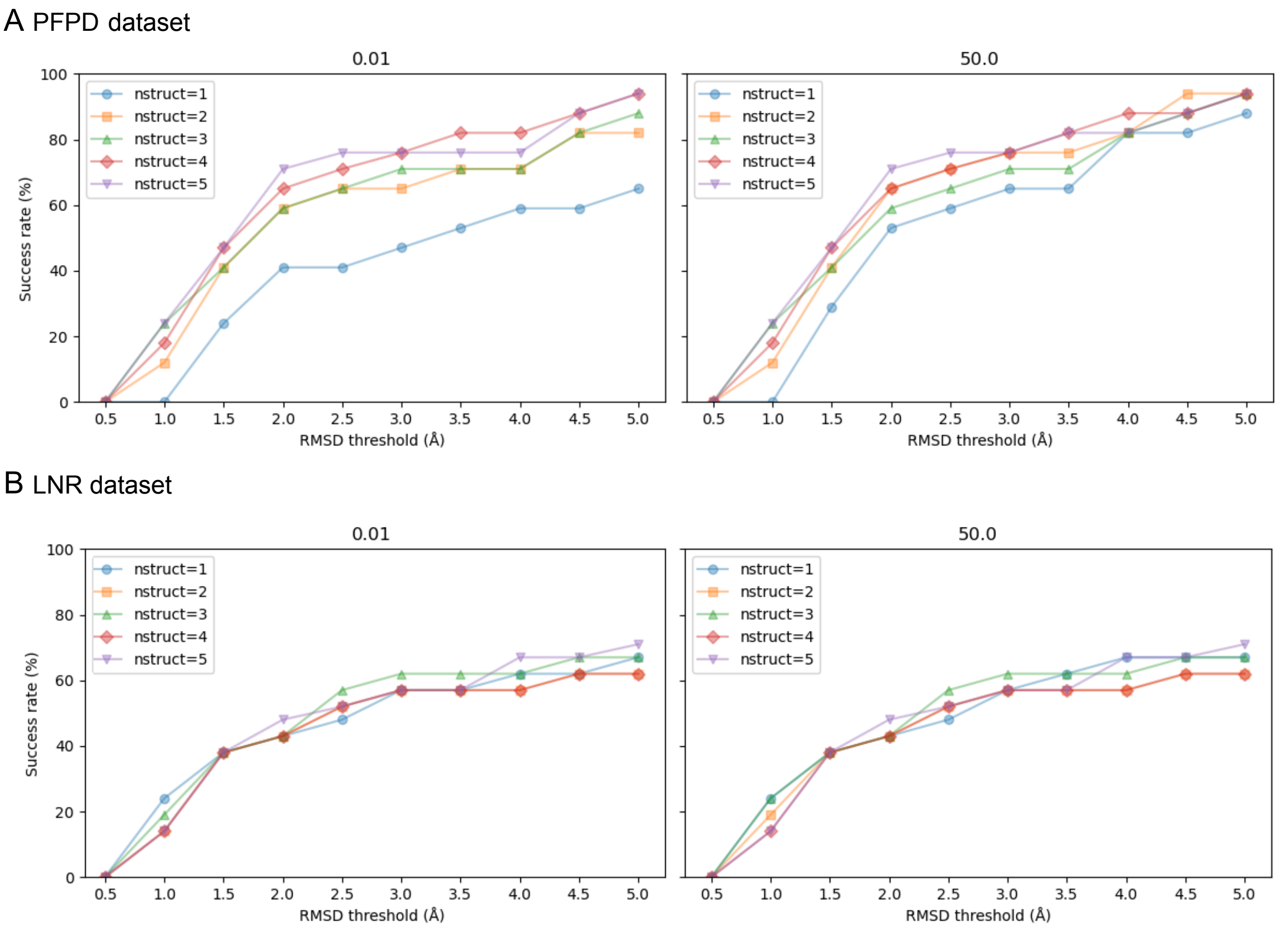

Figure S7. Calibration of number of refinement runs and decoys selected for clustering for focus runs. Results are shown for benchmark sets PFPD **(A)** and LNR (**B**). Decoys are selected for clustering according to a fixed fraction of the total decoy set (**right panels**), or a fixed number of decoys (**left panels**). One to five refinements per fragment are evaluated (see color code inset). The most significant improvement is observed for increasing *nstruct,* in particular increasing to *nstruct >1*, and for some further increasing to *nstruct >3*. **Accompanies Figure 3.**

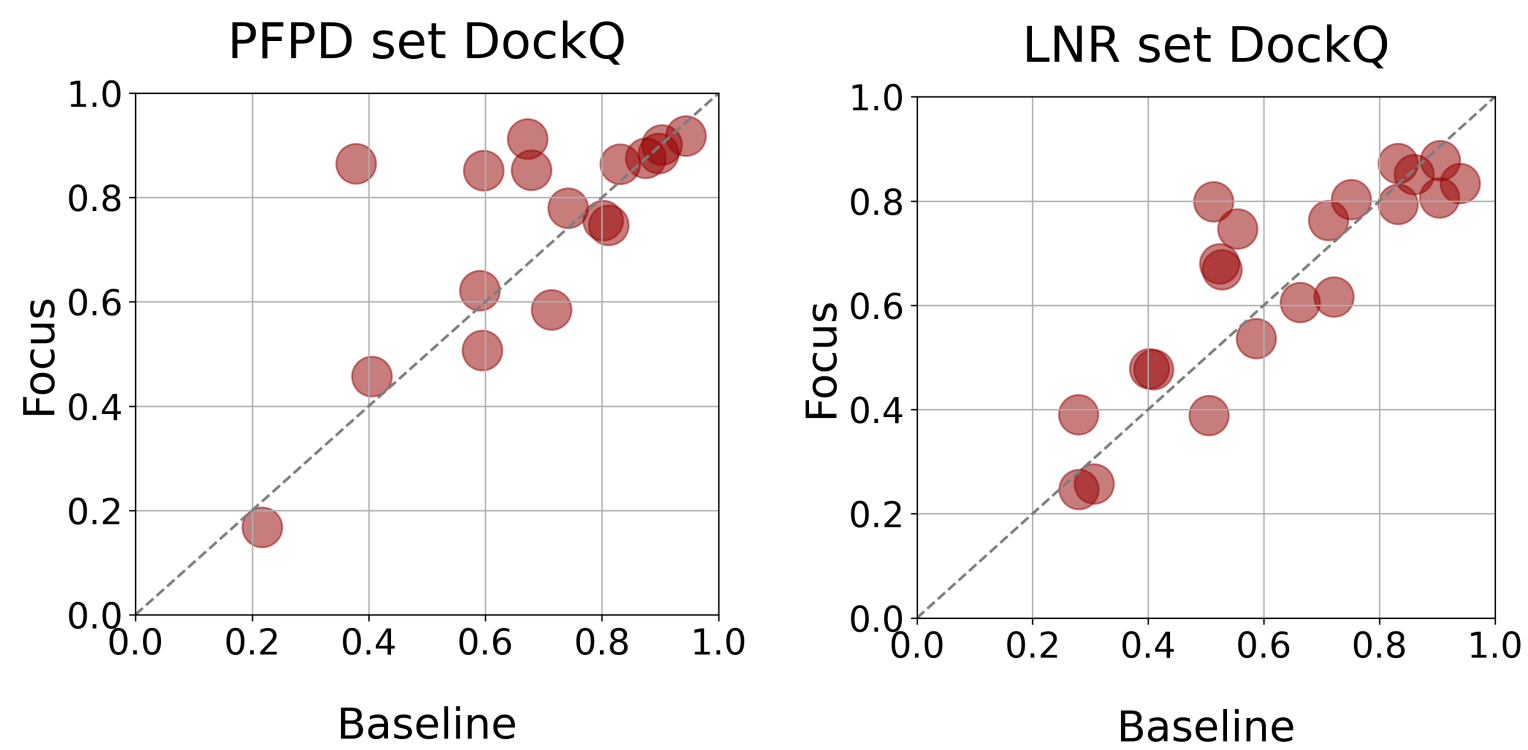

Figure S8. Scatter plot comparing docking performance obtained with PatchMAN2$_{baseline}$ and focus-guided PatchMAN2 on the PFPD (right) and LNR benchmark set (left). Each point represents one complex; performance is reported as the best DockQ value among the top 10 cluster representatives. The dashed line indicates equal performance between the two protocols. **Accompanies Figures 4A,C.**

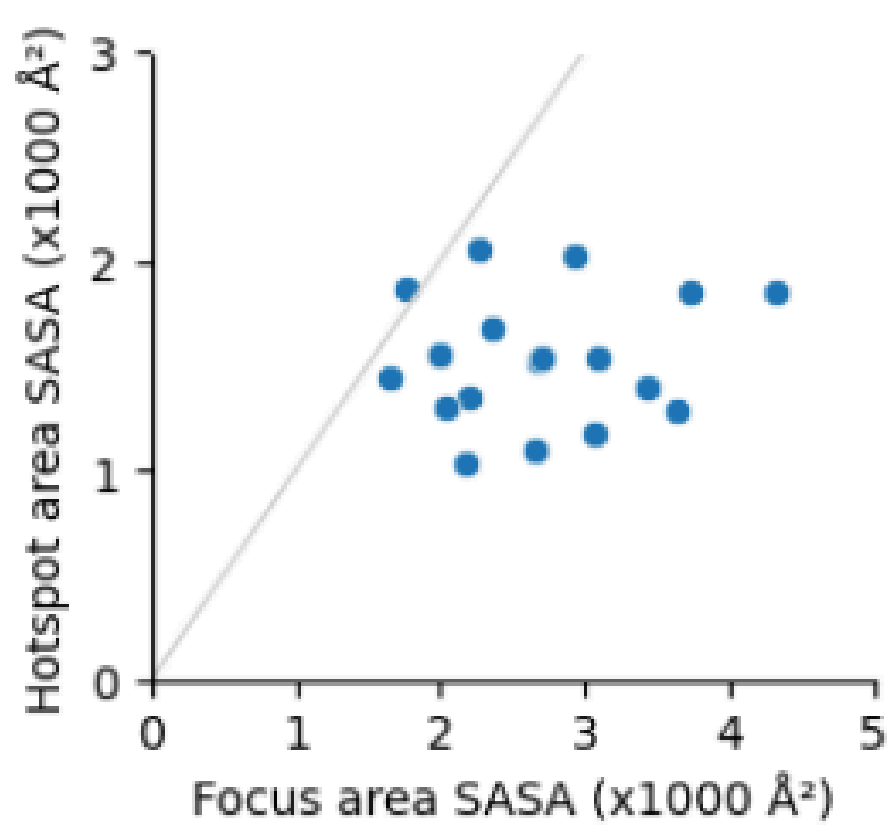

Figure S9. Comparison of receptor surface area covered by hotspot-defined input regions (after expansion; see Methods) and focus-defined regions. The gray line indicates equal surface area coverage.

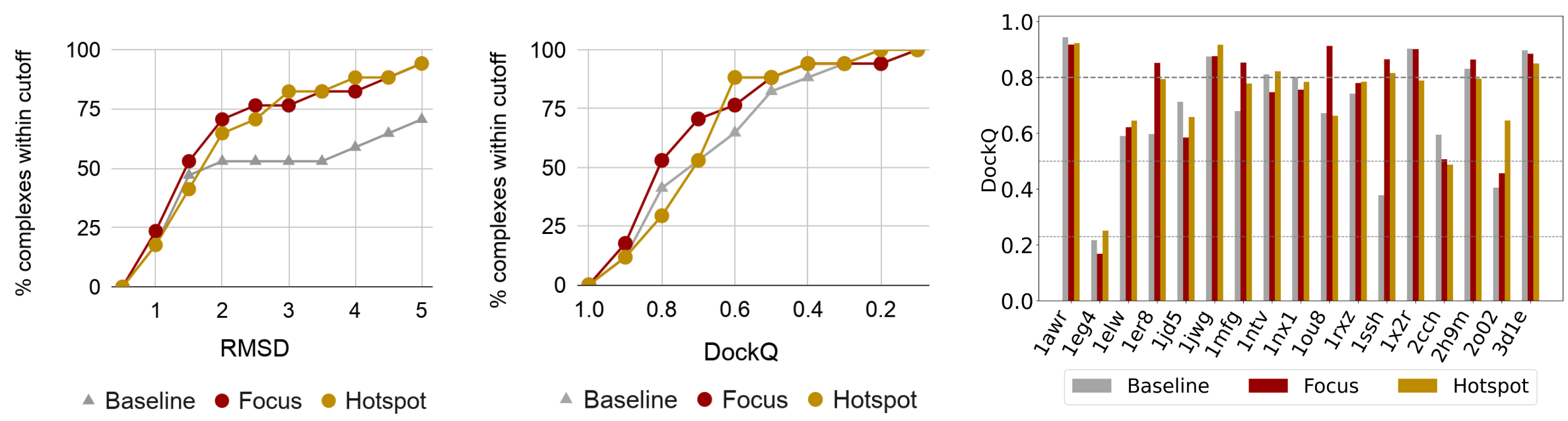

Figure S10. RMSD and DockQ assessment of performance for the PFPD set (n=17): Comparison of the PatchMAN2$_{baseline}$, Focus and Hotspot results. **Left and middle:** Cumulative plots of RMSD and DockQ values. Legend as in **Figure S1. Right**: Individual results. **Accompanies Figure 4E.**

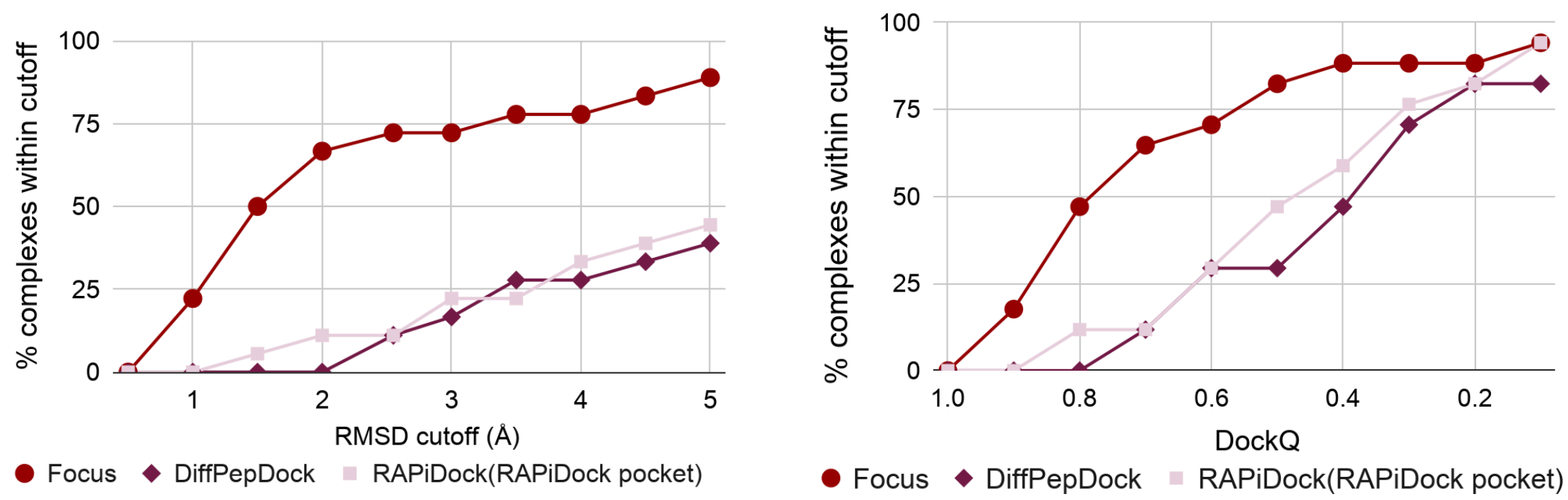

Figure S11. Performance of local docking runs for the PFPD set (n=17): Comparison of PatchMAN2 Focus, DiffPepDock and RAPiDock results, based on RMSD (**left**) and DockQ (**right**) measures. Legend as in **Figure S1.**

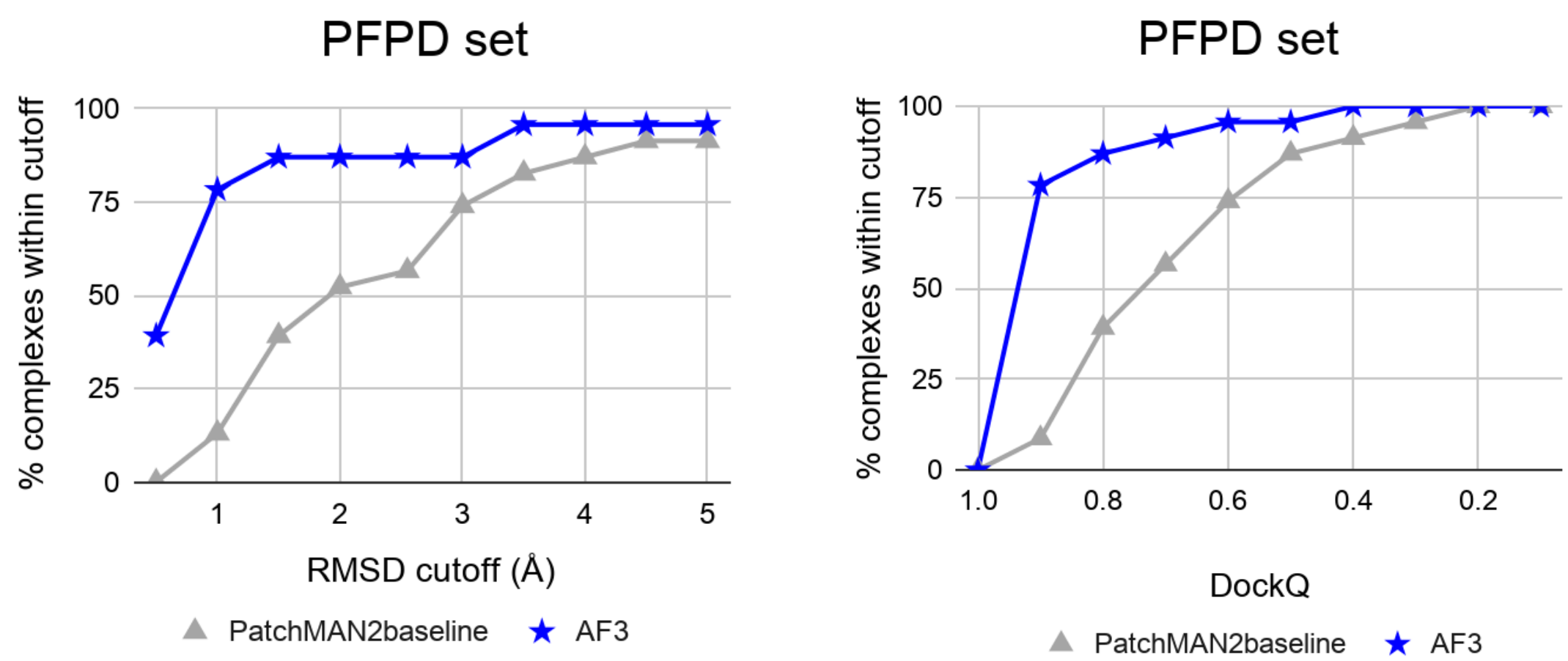

Figure S12. Performance of global docking for the PFPD set (n=23): Comparison of PatchMAN2$_{baseline}$ and AF3 results, based on RMSD (**left**) and DockQ (**right**) values. Legend as in **Figure S1.**

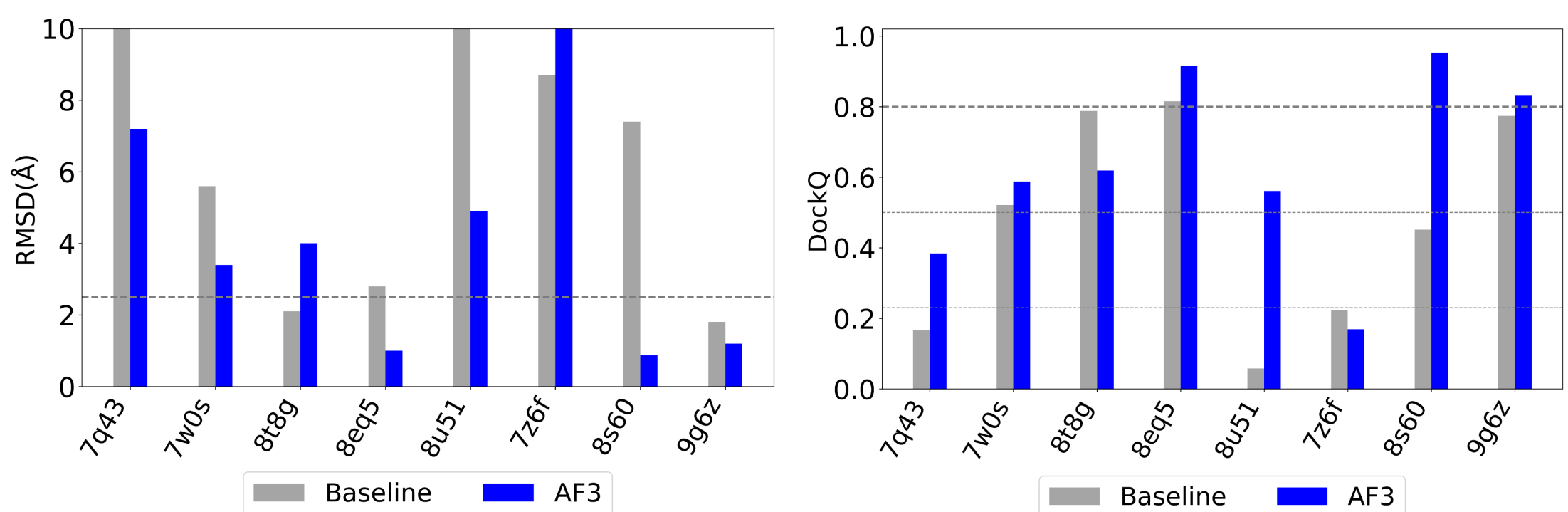

Figure S13. Performance of global docking on novel protein-peptide complex structures (n=8, see **Table S1C**). Comparison of PatchMAN2$_{baseline}$ and AF3 results. While PatchMAN2$_{baseline}$ performance is maintained, AF3 performance decreases significantly compared to predictions on complexes that it has seen during training (Compare to **Figure 1E** and **Table S3A**)

Table S1. **A,B.** The two benchmark sets used in this study, composed of complexes for which a suitable bound homologue structure is available (with a peptide located within 6 Å RMSD of the peptide in the benchmark structure). **A.** PFPD set; **B.** LNR set. **C.** Set of recently released peptide-receptor complexes not included for training of Alphafold3.

A. PFPD benchmark set (23 complexes with solved unbound structure; 17 with solved homologue bound structure)

**Complex pdb**	**ECOD**	**Unbound pdb**	**Homologue for focus**	**Focus size (# residues)**	**Hotspot residues**
1AWR:CI	75.1.1.1	2ALF	1CWB:A	25	62,54,59
1EG4:AP	64.1.1.1	1EG3	1F4V:A	28	24,131,13
1ELW:AC	109.4.1.1288	1A17	1H27:B	14	83,49,18
1ER8:EI	1.1.1.1	4APE	1NX0:B	44	79,222,219
1JD5:AB	381.1.1.1	1JD4	1OEB:A	15	72,68,73
1JWG:BD	109.4.1.15	1JWF	2QAS:A	20	86,96,82
1MFG:AB	7.1.1.1	2H3L	2QT5:B	20	34,27,83
1NTV:AB	220.1.1.6	1P3R	3G2S:A	26	91,134,108
1NX1:AC	108.1.1.0	1ALV	3UQR:A	26	73,35,80
1OU8:BD	4.1.2.1	1OU9	3UVK:A	22	28,75,53
1RXZ:AB	227.1.1.1	1RWZ	3UW5:B	34	241,44,242
1SSH:AB	4.1.1.1	1OOT	4CGW:B	22	38,54,17
1X2R:AB	5.1.3.6	1X2J	4TSZ:A	21	92,160,249
2CCH:DF	101.1.10.1	1H1R	5NJK:D	19	80,106,50
2H9M:CD	5.1.4.256	2H14	6EJL:B	37	229,18,102
2O02:AP	109.4.1.0	2BQ0	6K3A:C	31	124,173,42
3D1E:AP	227.1.1.3	3D1G	6KKG:A	28	175,247,362
1CZY:CE	11.10.1.4	1CA4			
1U00:AP	511.1.1.1	2V7Y			
2B9H:AC	206.1.1.1	2B9F			
2C3I:BA	206.1.1.71	2J2I			
2DS8:BP	377.1.1.17	2DS7			
2HPL:AB	320.4.1.3	2HPJ			

B. LNR benchmark set (39 subset with solved unbound structure: 21 with solved bound homologue structure; out of total 96 complexes)

Complex pdb	ECOD	Unbound pdb	Homologue for focus	Focus size (# residues)
1D4T:AB	214.1.1.1	1D1Z:A	1LKL:A	27
1T3L:AB	4.1.1.0	1T3S:A	1T0J:B	32
1T5Z:AB	188.1.1.1	1E3G:A	6KTM:A	27
1TJ9:AB	371.1.1.1	2QU9:A	1JQ9:A	29
2B1N:AB	219.1.1.1	2B1M:A	1MHW:B	14
2FIB:AB	269.1.1.1	3FIB:A	1FZF:F	21
2V8X:AB	317.1.1.1	5GW6:A	5EKV:A	24
2WV5:CG	1.1.17.2	2J92:A	3SJ9:A	31
2Z9I:AG	1.1.5.33	1Y8T:A	7CO5:F	40
3AYU:AB	2498.1.1.1	1QIB:A	4JQG:B	30
3C3O:AB	109.4.1.37	2OEW:A	5MK3:A	26
3N2D:AB	292.1.1.1	3RL9:A	2JDL:A	29
3N5U:AC	246.2.1.4	6G0J:A	2P6B:A	20
4BTA:AC	3777.1.1.1	4BT8:A	2V5F:A	19
4Z2O:AP	9.2.1.1	4Z27:A	1KL3:A	32
5N85:AB	2.1.1.29	4IPG:A	5EAY:B	27
5YC2:AB	101.1.9.0	5YBX:A	5YCA:A	23
6CCT:AB	9.2.1.2	6CCR:A	6WZX:A	19
6HGT:DH	10.12.1.10	2GP5:A	4HON:A	42
6J0X:AE	7568.1.1.0	6J0V:A	6J0W:A	52
6N3E:AB	304.9.1.1	6N3F:A	2PEH:B	21
1KY7	11.1.1.44	1QTS		
1P7W	2499.1.1.1	1EGQ		
1XQY	7579.1.1.6	1XQX		
2ORZ	10.32.1.2	2ORX		
3BRH	2007.2.3.1	2QCJ		
3DNJ	308.1.1.2	3G3P		
3NIH	904.2.1.1	3NIL		
3R42	216.1.1.3	3R3Q		
3R7G	206.1.1.31	2YLF		
3UFM	7569.1.1.1	2BOO		
4FVD	1.1.5.5	4FVB		
4MVK	9.1.1.1	6GQZ		
4TJX	2487.1.1.7	4TJV		
5GR9	207.1.1.131	5JFK		
5JIU	10.1.1.8	5JI9		
5ONP	2498.1.1.12	5DLH		
5SGA	1.1.17.1	2SGA		
6FC6	4.1.1.9	6FC5		

C. Set of recently released peptide-receptor complexes not included for training of Alphafold3 (8 complexes released after 9/2021)

Complex pdb	Unbound pdb/Alphafold2 model*
7Q43	7Q40
7W0S	6UMB
8T8G	3FN5
8EQ5	8XFY
8U51	8U50
7Z6F	AF-P33913-F1
8S6O	AF-A0A0D1DWZ5-F1
9G6Z	AF-Q09293-F1

* from the AlphaFold Protein Structure Database AFDB(1).

Table S2. Definition of BSA cutoff for different peptide lengths

Peptide length	Minimum BSA (freeSASA*) [Å^2]	Cutoff (freeSASA) [Å^2]	Minimum BSA (pyRosetta**) [Å^2]	Cutoff (pyRosetta) [Å^2]
3	185	100	239	200
4	210	200	279	200
5	326	200	405	200
6	222	200	291	200
7	332	300	384	300
8	412	300	473	400
9	406	300	457	400
10	386	300	541	500
11	466	400	518	500
12	554	400	599	500
13	484	400	516	500
14	560	500	610	600
15	552	500	625	600
16	647	600	717	600
17	789	600	823	600
18	690	600	721	600
19	616	600	699	600
20	780	700	830	800
21	967	900	1083	1000
27	959	900	1055	1000

* as calculated using FreeSASA(2)
** as calculated using PyRosetta(3)

Table S3. Runtime comparison across CPU nodes (for 1AWR as example).

**CPU model**	**Architecture**	**Clock (GHz)**	**Median time/decoy (min)**
Intel Xeon E5-2670 v2	Ivy Bridge-EP (AVX)	2.5	5.1
Intel Xeon E5-2643	Sandy Bridge-EP (AVX)	3.3	5.7
Intel Xeon E5-2650 v4	Broadwell-EP (AVX2)	2.2	6.7
Intel Xeon X5550	Nehalem-EP (no AVX)	2.67	8.1

Table S4. Performance of PatchMAN after addition of BSA filter. Comparison of PatchMAN with PatchMAN2 and AF3. Significantly improved and worse cases are highlighted in bold and italics, respectively.

A. PFPD set

Complex pdb	RMSD*				DockQ			
	PatchMAN			AF3	PatchMAN			AF3
	Original (PatchMAN)	PatchMAN2 (BSA Top 1%)	PatchMAN$_{baseline}$ (BSA Top 50)		Original (PatchMAN)	PatchMAN2 (BSA Top 1%)	PatchMAN2$_{baseline}$ (BSA Top 50)	
1AWR:CI	0.9	0.8	0.8	0.8	0.93	0.93	0.94	0.96
1CZY:CE	2.8	2.8	2.8	**0.65**	0.78	0.78	0.78	**0.98**
1EG4:AP	19.6	9.2	9.2	**1.5**	0.12	0.22	0.22	**0.83**
1ELW:AC	2.0	**1.6**	**1.6**	**0.4**	0.60	0.60	0.59	**0.98**
1ER8:EI	9.7	10.3	**3.7**	**0.6**	0.29	0.24	0.60	**0.94**
1JD5:AB	3.7	**2.7**	**2.7**	**0.25**	0.59	0.71	0.71	0.66 (0.94)
1JWG:BD	1.4	1.3	1.3	1.04	0.88	0.88	0.88	0.94
1MFG:AB	9.4	7.1	**2.5**	**0.7**	0.27	0.27	0.68	0.98
1NTV:AB	1.2	1.2	1.2	**0.4**	0.81	0.81	0.81	0.98
1NX1:AC	11.3	**1.2**	1.2	**0.4**	0.20	**0.80**	**0.80**	0.97
1OU8:BD	4.2	4.2	**2.6**	**0.3**	0.59	0.59	0.67	**0.99**
1RXZ:AB	1.1	1.1	1.1	0.8	0.74	0.74	0.74	**0.89**
1SSH:AB	**1.3**	*5.2*	**3.3**	**0.4**	0.85	0.38	0.64	**0.99**
1U00:AP	2.0	2.0	2.0	**0.5**	0.36	0.36	0.36	**0.96**
1X2R:AB	0.9	0.9	0.9	1	0.90	0.90	0.90	0.94
2B9H:AC	3.4	3.4	3.4	**0.75**	0.62	0.62	0.65	**0.93**
2C3I:BA	3.7	**2.6**	**2.6**	**0.3**	0.61	0.61	0.74	**0.96**
2CCH:DF	4.9	4.2	4.2	**0.3**	0.50	0.60	0.60	**0.94**
2DS8:BP	1.3	1.3	1.3	**0.6**	0.57	**0.82**	**0.82**	0.93
2H9M:CD	1.3	1.3	1.3	**0.7**	0.87	0.87	0.83	0.95
2HPL:AB	1.7	1.7	1.7	3.2	0.87	0.87	0.87	0.98
2O02:AP	9.2	**5.5**	**5.5**	3.2	0.23	0.41	0.41	**0.72**
3D1E:AP	1.0	1.0	1.0	*6.4*	0.90	0.90	0.90	*0.44*

* RMSD values (in [Å], calculated over peptide interface residue backbone atoms) for the best prediction among representatives of the top 10 clusters, as in Khramushin *et al.(4)*

B. **LNR set.** Legend as in **A**

Complex pdb	PatchMAN					
	RMSD			DockQ		
	Original (PatchMAN)	PatchMAN2 (BSA Top1%)	PatchMAN2$_{baseline}$ (BSA Top50)	Original (PatchMAN)	PatchMAN2 (BSA Top1%)	PatchMAN2$_{baseline}$ (BSA Top50)
1D4T	8.3	9.8	9.8	0.3	0.3	0.3
1KY7	15	15.5	15.5	0.1	0.1	0.1

Complex pdb	PatchMAN					
	RMSD			DockQ		
	**Original (PatchMAN)**	**PatchMAN2** (BSA Top1%)	**PatchMAN2$_{baseline}$** (BSA Top50)	**Original (PatchMAN)**	**PatchMAN2** (BSA Top1%)	**PatchMAN2$_{baseline}$** (BSA Top50)
1P7W	4.2	4.2	4.2	0.7	0.7	0.7
1T3L	1.4	1.4	1.4	0.8	0.8	0.8
1T5Z	1.4	1.4	1.4	0.9	0.9	0.9
1TJ9	4.9	*6.1*	*6.1*	0.6	0.5	0.5
1XQY	2.9	2.9	2.9	0.7	0.7	0.7
2B1N	1.4	1.4	1.4	0.9	0.9	0.9
2FIB	2.1	2.0	2.0	0.8	0.8	0.8
2ORZ	4.1	*27.5*	*27.5*	0.7	0.0	0.0
2V8X	0.9	0.8	0.8	0.9	0.9	0.9
2WV5	6.3	6.3	6.3	0.4	0.4	0.4
2Z9I	0.8	0.8	0.8	0.9	0.9	0.9
3AYU	2.8	2.7	2.7	0.7	0.7	0.7
3BRH	8.8	9.9	9.9	0.4	0.2	0.2
3C3O	4.7	*5.9*	*5.9*	0.5	0.6	0.6
3DNJ	0.8	0.8	0.8	0.9	0.9	0.9
3N2D	9.6	9.6	9.6	0.3	0.3	0.3
3N5U	2.9	*3.2*	*3.2*	**0.8**	0.7	0.7
3NIH	1.0	1.0	1.0	0.2	**0.9**	**0.9**
3R42	13.9	14.6	14.6	0.0	0.1	0.1
3R7G	10.2	11.0	11.0	0.3	0.3	0.3
3UFM	4.6	4.6	4.6	0.6	0.6	0.6
4BTA	3.6	*6.7*	*6.7*	0.5	0.5	0.5
4FVD	1.9	1.9	1.9	0.9	0.9	0.9
4MVK	2.4	2.4	2.4	0.5	0.5	0.5
4TJX	16.7	16.7	16.7	0.0	0.0	0.0
4Z2O	1.7	1.8	1.8	0.8	0.8	0.8
5GR9	6.3	6.1	6.1	0.5	0.5	0.5
5JIU	7.4	7.4	7.4	0.4	0.4	0.4
5N85	7.7	6.0	6.0	0.4	0.4	0.4
5ONP	21.9	21.9	21.9	0.0	0.1	0.1
5SGA	0.3	0.3	0.3	1.0	1.0	1.0
5YC2	1.1	*2.4*	*2.4*	0.7	0.7	0.7
6CCT	4.8	4.8	4.8	0.5	0.5	0.5
6FC6	5.3	5.3	5.3	0.6	0.6	0.6
6HGT	5.3	5.3	5.3	0.5	0.5	0.5
6J0X	15.2	9.4	9.4	0.3	0.3	0.3
6N3E	1.3	1.3	1.3	0.9	0.9	0.9

Table S5. **Masking the obligatory interface of homomultimer complexes improves PatchMAN results.** Shown are RMSD (in [Å], calculated over peptide interface residue backbone atoms) and DockQ values for the best prediction among representatives of the top 10 clusters.

Complex pdb	RMSD		DockQ	
	Masked	Non-masked	Masked	Non-masked
1CZY	**1.4**	*2.8*	**0.88**	0.78
1NX1	1.3	1.2	0.87	0.80
1OU8	**0.5**	*2.6*	**0.98**	0.67
1RXZ	1.45	1.1	**0.89**	0.74
2DS8	1.9	1.3	0.80	0.82
2O02	**3.20**	5.5	0.66	0.41
4BTA	**1.33**	6.7	**0.86**	0.51

Table S6. **Effect of focus filters on performance** (PFPD benchmark set). Shown are results where a bound homologue structure is available (**Table S1**): the best prediction among representatives of the top 10 clusters (RMSD values in [Å], calculated over peptide interface residue backbone atoms).

Complex pdb	$\text{PatchMAN2}_{baseline}$	Filter 1 $N_{focus\_overlap} \geq 5$	Filter 2 $N_{receptor\_focus\_contacts} \geq 3$	Focus: Filters 1&2
1AWR	0.8	0.8	0.8	0.8
1EG4	9.2	6.3	**4.9**	6.3
1ELW	1.6	**1.3**	1.6	**1.3**
1ER8	3.7	3.7	3.7	3.7
1JD5	2.7	2.5	2.7	2.5
1JWG	1.3	1.3	1.3	1.3
1MFG	2.5	**1.8**	**1.8**	**1.8**
1NTV	1.2	1.2	1.2	1.2
1NX1	1.2	1.2	1.2	1.2
1OU8	2.6	4.3	2.6	3.1
1RXZ	1.1	1.5	1.1	1.5
1SSH	3.3	3.3	3.3	3.3
1X2R	0.9	*3.7*	0.9	*3.7*
2CCH	4.2	4.3	4.2	4.3
2H9M	1.3	1.3	1.3	1.3
2O02	5.5	5.5	5.5	5.5
3D1E	1.0	1.0	1.0	1.0

Table S7. Results of focus, hotspot, DiffPepDock and RAPiDock runs for complexes in the PFPD and LNR benchmark sets, where applicable (*i.e.,* a homolog bound structure is available, see **Table S1**). Shown are RMSD values (in [Å], calculated over peptide interface residue backbone atoms) for the best prediction among representatives of the top 10 clusters. For comparison, we also show the results of *RAPiDock* and *DiffPepDock* simulations, assessed based on recommended parameters (*RAPiDock:* best prediction among 10 models; *DiffPepDock*: best prediction among the top 23 models).

A. PFPD set

Complex PDB	RMSD					DockQ				
	PatchMAN2$_{baseline}$	Focus	Hotspot	DiffPepDock	RAPiDock	PatchMAN2$_{baseline}$	Focus	Hotspot	DiffPepDock	RAPiDock
1AWR	0.8	1.3	1.1	2.3	*8.7*	0.94	0.92	0.92	0.76	0.45
1EG4	9.2	10.3	9.8	*21.1*	*10.2*	0.22	0.17	0.25	0.06	0.26
1ELW	1.6	1.5	1.2	*7.8*	*6.9*	0.59	0.62	0.65	0.26	0.12
1ER8	3.7	**0.9**	**1.0**	*6.6*	*9.9*	0.60	**0.85**	0.79	0.50	0.69
1JD5	2.7	*3.4*	**2.8**	*8.8*	*5.7*	0.71	0.59	0.66	0.28	0.52
1JWG	1.3	**0.7**	**0.6**	*3.2*	*7.3*	0.88	0.88	0.92	0.70	0.53
1MFG	2.5	**1.2**	**1.5**	*7.1*	*8.2*	0.68	**0.85**	0.78	0.41	0.58
1NTV	1.2	*1.8*	**0.8**	*6.6*	*12.3*	0.81	0.75	**0.82**	0.38	0.37
1NX1	1.2	1.6	1.6	*3.2*	*5.7*	0.80	0.76	0.78	0.67	0.70
1OU8	2.6	**1.2**	*3.8*	*6.8*	*5.4*	0.67	**0.91**	0.66	0.33	0.62
1RXZ	1.1	*2.2*	**1.7**	*5.0*	*9.7*	0.74	0.78	0.79	0.50	0.46
1SSH	3.3	**1.3**	**1.5**	*4.9*	*6.8*	0.87	0.87	0.82	0.39	0.38
1X2R	0.9	0.7	*2.0*	2.2	*6.6*	0.90	0.90	0.79	0.66	0.86
2CCH	4.2	4.8	4.6	*7.4*	*10.0*	0.60	0.51	0.49	0.31	0.32
2H9M	1.3	1.8	*2.1*	3.5	*6.0*	0.83	0.86	0.80	0.66	0.85
2O02	5.5	4.3	**2.8**	*16.2*	*13.1*	0.41	0.46	0.65	0.10	0.13
3D1E	1.0	0.8	1.7	*7.4*	3.1	0.90	0.89	0.85	0.34	0.47

B. LNR set. Legend as in A

Complex PDB	RMSD		DockQ	
	PatchMAN2$_{baseline}$	Focus	PatchMAN2$_{baseline}$	Focus
1D4T	9.8	11.1	0.3	0.2
1T3L	1.4	1.4	0.8	0.8
1T5Z	1.4	1.4	0.9	0.8
1TJ9	6.1	6.8	0.5	0.4
2B1N	1.4	**0.7**	0.9	0.9
2FIB	2.0	**0.6**	0.8	0.8

2V8X	0.8	0.9	0.9	0.8
2WV5	6.3	5.8	0.4	0.5
2Z9I	0.8	0.9	0.9	0.9
3AYU	2.7	**1.2**	0.7	0.6
3C3O	5.9	**2.5**	0.6	0.7
3N2D	9.6	9.0	0.3	0.4
3N5U	3.2	**2.8**	0.7	**0.8**
4BTA	6.7	**2.2**	0.5	**0.8**
4Z2O	1.8	**0.8**	0.8	0.9
5N85	6.0	5.5	0.4	0.5
5YC2	2.4	2.5	0.7	0.6
6CCT	4.8	5.0	0.5	0.7
6HGT	5.3	4.2	0.5	0.7
6J0X	9.4	*12.0*	0.3	0.3
6N3E	1.3	1.7	0.6	0.5

Table S8. Results of global runs of PatchMAN2$_{baseline}$ *vs.* AF3 on the set of 8 novel peptide-receptor complexes (**Table S1C**). Shown are RMSD values (in [Å], calculated over peptide interface residue backbone atoms) and DockQ values, for the best prediction among representatives of the top 10 clusters for Patchman and best among the 5 models.

Complex PDB	RMSD		DockQ	
	PatchMAN2$_{baseline}$	AF3	PatchMAN2$_{baseline}$	AF3
7Q43	11.9	7.2	0.17	0.38
7W0S	*5.6*	3.5	0.52	0.59
8T8G	**2.1**	*4.3*	0.79	0.62
8EQ5	*2.8*	**0.9**	0.82	0.92
8U51	*22*	4.8	0.06	0.56
7Z6F	8.7	12.3	0.22	0.17
8S6O	*7.4*	**0.9**	0.45	**0.95**
9G6Z	**1.8**	**1**	0.77	**0.83**

References

1. Varadi M, Bertoni D, Magana P, Paramval U, Pidruchna I, Radhakrishnan M, et al. AlphaFold Protein Structure Database in 2024: providing structure coverage for over 214 million protein sequences. Nucleic Acids Res. 2024 Jan 5;52(D1):D368–75.
2. Mitternacht S. FreeSASA: An open source C library for solvent accessible surface area calculations. [version 1; peer review: 2 approved]. F1000Res. 2016 Feb 18;5:189.
3. Chaudhury S, Lyskov S, Gray JJ. PyRosetta: a script-based interface for implementing molecular modeling algorithms using Rosetta. Bioinformatics. 2010 Mar 1;26(5):689–91.
4. Khramushin A, Ben-Aharon Z, Tsaban T, Varga JK, Avraham O, Schueler-Furman O. Matching protein surface structural patches for high-resolution blind peptide docking. Proc Natl Acad Sci USA. 2022 May 3;119(18):e2121153119.
